# Clinical Outcomes of Intravenous Thrombolysis in Acute Ischemic Stroke: A Prospective Observational Study From Mumbai

**DOI:** 10.7759/cureus.92011

**Published:** 2025-09-10

**Authors:** Vivek Solapure, Sandeep Gore, Keyur Shah, Pradip Shah

**Affiliations:** 1 Emergency Medicine, Fortis Hospital, Mulund, IND; 2 Emergency Medicine, Pramukhswami Medical College, Anand, IND; 3 Internal Medicine, Fortis Hospital, Mulund, IND

**Keywords:** india, ischemic stroke, iv thrombolysis, recombinant tissue plasminogen activator (rt-pa), stroke

## Abstract

This prospective observational study was conducted to evaluate the clinical outcomes of intravenous thrombolysis using recombinant tissue plasminogen activator (rt-PA) in patients with acute ischemic stroke at a tertiary care center in Mumbai, India. A total of 54 eligible patients received intravenous rt-PA within 4.5 hours of symptom onset. The primary outcome was neurological improvement assessed by the National Institutes of Health Stroke Scale (NIHSS). The mean door-to-needle time was 54.2±21.2 minutes, with 77.77% of patients receiving treatment within 60 minutes of hospital arrival. A significant reduction in NIHSS scores was observed from baseline (9.09±3.69) to day seven post-treatment (2.27±1.96), with a p-value of <0.001, indicating substantial neurological recovery. There were three deaths recorded during the study period. The findings underscore the effectiveness of timely rt-PA administration in improving neurological outcomes in acute ischemic stroke. Optimizing door-to-needle time through streamlined acute stroke protocols remains essential to enhance therapeutic success in resource-constrained healthcare settings.

## Introduction

Acute stroke is characterized by the sudden onset of focal neurological deficits corresponding to a specific vascular territory within the brain, retina, or spinal cord, resulting from underlying cerebrovascular pathology [1,2]. It remains the third leading cause of mortality worldwide [3]. Acute ischemic stroke occurs due to thromboembolic occlusion of one or more cerebral arteries, leading to acute neurological impairment. At the cellular level, ischemia induces adenosine triphosphate (ATP) depletion secondary to hypoxia, which disrupts ion homeostasis and triggers neuronal depolarization [4]. Ischemic strokes comprise approximately 87% of all stroke cases globally [2].

The administration of intravenous thrombolytic therapy has been demonstrated to be effective in restoring cerebral perfusion and reversing neurological deficits in patients with acute ischemic stroke [5]. Tissue plasminogen activator (t-PA), an endogenous serine protease produced by vascular endothelial cells, plays a key role in fibrinolysis by converting plasminogen to plasmin, which subsequently degrades fibrin and dissolves thrombi. Recombinant tissue plasminogen activator (rt-PA), commercially known as alteplase, closely mimics the endogenous form but exhibits enhanced fibrin specificity [6]. Alteplase is more resistant to inactivation by plasminogen activator inhibitor-1 (PAI-1), enabling prolonged thrombolytic activity through sustained binding to fibrin within the thrombus. The efficacy of fibrinolysis is influenced by several factors, including local blood flow dynamics and thrombus burden. As fibrinolysis progresses, microstreaming of alteplase through the clot, facilitated by arterial pulsations, leads to the gradual and complete dissolution of the thrombus [7].

Timely administration of intravenous alteplase is critical to maximizing therapeutic benefit. Current guidelines recommend its use within three hours of symptom onset, with an extended window up to 4.5 hours in selected patients [8]. Although the efficacy of rt-PA therapy is well documented in global studies, limited data are available from the Indian context. Therefore, this study aimed to evaluate the clinical outcomes following intravenous administration of rt-PA within the recommended therapeutic window (up to 4.5 hours) in patients with acute ischemic stroke presenting to a tertiary care center in Mumbai.

## Materials and methods

This prospective observational study was conducted in the Emergency Department and Intensive Care Unit of Fortis Hospital, Mulund, Mumbai, India, between March 2017 and August 2018.

Patients presenting with symptoms suggestive of acute ischemic stroke were systematically assessed and investigated, with all critical time points documented across five key stages: (1) door to initial physician assessment, (2) time since symptom onset, (3) door to neuroimaging, (4) door to imaging interpretation, and (5) door to initiation of intravenous thrombolysis (door-to-needle time), all measured in minutes. Clinical evaluation included calculation of the baseline National Institutes of Health Stroke Scale (NIHSS) score to quantify stroke severity [9]. Neuroimaging, either magnetic resonance imaging (MRI) of the brain using a diffusion-weighted protocol or non-contrast computed tomography scan (CT-scan) of the brain, was performed promptly to guide diagnosis. Based on clinical and imaging findings, patients were screened using the predefined standard inclusion and exclusion criteria to determine eligibility for intravenous tPA [8]. Intravenous thrombolysis with rt-PA was administered only to those patients who met all eligibility requirements.

Eligible patients received intravenous alteplase at a dose of 0.9 mg/kg, with 10% administered as an intravenous bolus over one minute and the remaining 90% infused over 60 minutes. The outcome of thrombolysis was assessed based on changes in NIHSS scores from baseline, with a reduction in score indicating clinical improvement and an increase suggesting deterioration. NIHSS scores were recorded at 1 hour, 24 hours, the third day, and seven days post-thrombolysis.

A total of 57 patients presenting with acute ischemic stroke and eligible for intravenous thrombolysis, based on the predefined inclusion and exclusion criteria for rt-PA administration, were initially identified during the study period [8]. However, three patients were excluded due to the non-availability of informed consent. Consequently, 54 patients were included in the final analysis.

All data were recorded in an MS Excel spreadsheet (Microsoft® Corp., Redmond, WA) and analyzed using IBM SPSS Statistics, version 26 (IBM Corp., Armonk, NY). Qualitative data were presented as frequency and percentage, while continuous data were presented as mean ± SD. Comparison of mean scores was conducted using ANOVA, and a p-value <0.05 was considered statistically significant.

The study received approval from the Institutional Ethics Committee. Prior to enrollment, all participants were informed about the purpose of the study, and written informed consent was obtained. The procedures, techniques, and drugs employed in this study were part of standard clinical practice and routinely used in patient care. Therefore, there was no additional burden on either the patients or the institution. No treatment was withheld, and all clinical management was carried out in accordance with established guidelines.

## Results

The majority of patients were aged over 60 years, followed by those in the 41-60 years age group (37.0%), and a smaller proportion in the 26-40 years age group (7.4%). Males constituted a larger proportion of the study population compared to females. Regarding the duration of symptoms prior to hospital presentation, 35.8% of patients arrived within an hour, 26.4% (14/54) within 61-120 minutes, 24.5% (13/54) within 121-180 minutes, and 13.2% (7/54) within 181-240 minutes (Table 1). The mean duration of symptoms before presentation was 106.0±60.1 minutes.

**Table 1:** Baseline characteristics of patients (n=54)

Variables	Sub-group	Frequency	Percentage
Age-group (in years)	26-40	4	7.4%
	41-60	20	37.0%
	>60	30	55.6%
Gender	Female	15	27.8%
	Male	39	72.2%
Duration of symptoms (in minutes)	15-60	19	35.9%
	61-120	14	26.4%
	121-180	13	24.5%
	181-240	7	13.2%

The mean NIHSS score among the patients at admission or baseline was 9.09±3.69, with a range of five to 19. The majority (90.75%) had NIHSS scores between five and 15 (n=49), while 9.25% had scores ranging from 16 to 20 (n=5). The mean door-to-imaging time was 16.3 minutes (range: 2 to 40 minutes), and the mean door-to-imaging interpretation time was 27.1 minutes (range: 10 to 50 minutes). The mean door-to-needle time, representing the interval from hospital arrival to initiation of intravenous thrombolysis, was 54.2±21.2 minutes. The distribution of door-to-needle times was as follows: one patient (1.8%) received thrombolysis within 15-20 minutes, 15 patients (27.7%) within 21-40 minutes, 26 patients (48.1%) within 41-60 minutes, nine patients (16.6%) within 61-90 minutes, and three patients (5.5%) within 91-120 minutes. Notably, 42 out of 54 patients (77.77%) were thrombolysed within 60 minutes of arrival, aligning with the internationally recommended benchmark for timely acute stroke management.

**Figure 1:**
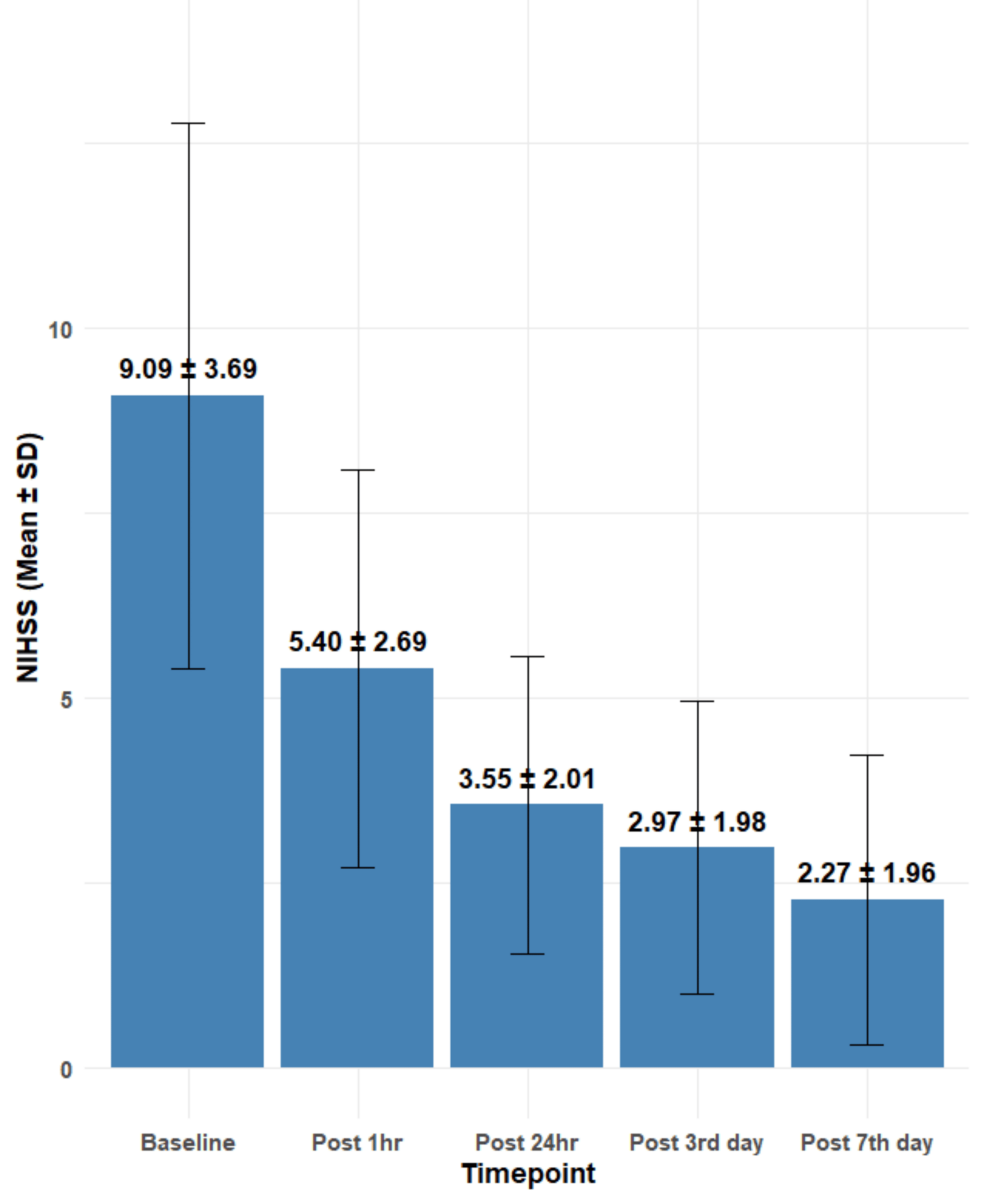
Comparison of NIHSS score over time NIHSS: National Institutes of Health Stroke Scale

Among the 54 cases, three mortalities were recorded. Two occurred within 24 hours following rt-PA therapy, and one occurred on the sixth day post-therapy. The mean NIHSS score at baseline was 9.09±3.69, which progressively declined over time following thrombolytic therapy. At one hour post-treatment, the mean NIHSS score reduced to 5.4±2.69, followed by further reductions at 24 hours (3.55±2.01), on the third day (2.97±1.98), and by the seventh day (2.27±1.96) (Figure 1). The change in NIHSS scores from baseline to the seventh day was statistically significant (F=60.31; df=4258; p<0.001), indicating a meaningful clinical improvement in neurological status over the observed period.

## Discussion

Over the past decade, the management of ischemic stroke has undergone significant advancements. Endovascular treatment has notably transformed therapeutic approaches. This study assessed the clinical outcomes of intravenous thrombolysis using rt-PA in patients with acute ischemic stroke at a tertiary care center in Mumbai, India. The results indicate substantial improvements in neurological function following rt-PA administration, as evidenced by progressive reductions in NIHSS scores over time.

The baseline characteristics of patients align with established epidemiological patterns of stroke, with a predominance of males and individuals over 60 years of age [10,11]. The mean duration from symptom onset to hospital arrival, recorded at 106 minutes, suggests a relatively prompt presentation. Similar findings have been reported by Khan et al., facilitating a significant proportion of patients to receive treatment within the recommended therapeutic window [11]. A notable strength of this study is the attainment of a mean door-to-needle time of 54.2 minutes, with 77.77% (42/54) of patients receiving thrombolysis within 60 minutes of arrival. This performance is closely aligned with international standards for acute stroke care and exceeds the outcomes reported in some prior studies [12,13]. The prompt initiation of treatment likely contributed to the favorable outcomes observed. In contrast, Khan et al. reported achieving a door-to-needle time of less than 60 minutes in only 41.2% of cases [11]. Additionally, a study from Coimbatore reported a mean door-to-needle time of 54.42 minutes, with a median of 50 minutes. In four cases, the door-to-needle time exceeded 60 minutes due to delays in obtaining consent for intravenous thrombolysis after explaining the advantages and disadvantages of intravenous rt-PA [14].

The progressive decline in mean NIHSS scores from baseline to seven days post-treatment demonstrates a clinically and statistically significant improvement in neurological status. This trend is consistent with findings from landmark trials such as the NINDS rt-PA Stroke Study, which reported that patients treated with t-PA were more likely to have minimal or no disability at three months compared to placebo [15]. Our results corroborate the findings of Wardlaw et al.’s systematic review, which showed significant improvements in clinical outcomes when rt-PA was administered within six hours of stroke onset, with the greatest benefit observed within three hours [16]. The majority of our patients received treatment well within this optimal timeframe, likely contributing to the positive outcomes observed.

While our findings are promising, it is important to acknowledge several limitations. The single-center design and relatively small sample size may restrict the generalizability of our results. Furthermore, the absence of a control group prevents direct comparisons with patients who did not receive thrombolytic therapy. Detailed information regarding potential complications of rt-PA, such as hemorrhagic transformation, was not systematically recorded. Additionally, only short-term outcomes were measured.

## Conclusions

This study demonstrates that the timely administration of intravenous rt-PA in eligible acute ischemic stroke patients results in significant neurological improvement, as measured by NIHSS scores. The findings underscore the importance of streamlined acute stroke protocols to minimize door-to-needle times and maximize the potential benefits of thrombolytic therapy. Further research is warranted to optimize stroke care pathways and improve outcomes for patients across diverse healthcare settings in India.
